# Supplementary material for: Arabidopsis FHY3 and FAR1 integrate light and strigolactone signaling to regulate branching
Source: Nat Commun. 2020 Apr 23;11:1955. doi: 10.1038/s41467-020-15893-7 (PMC7181604; doi:10.1038/s41467-020-15893-7)
Supplement: Supplementary file 1 — Supplementary Information [file 41467_2020_15893_MOESM1_ESM.pdf]

Supplementary figures

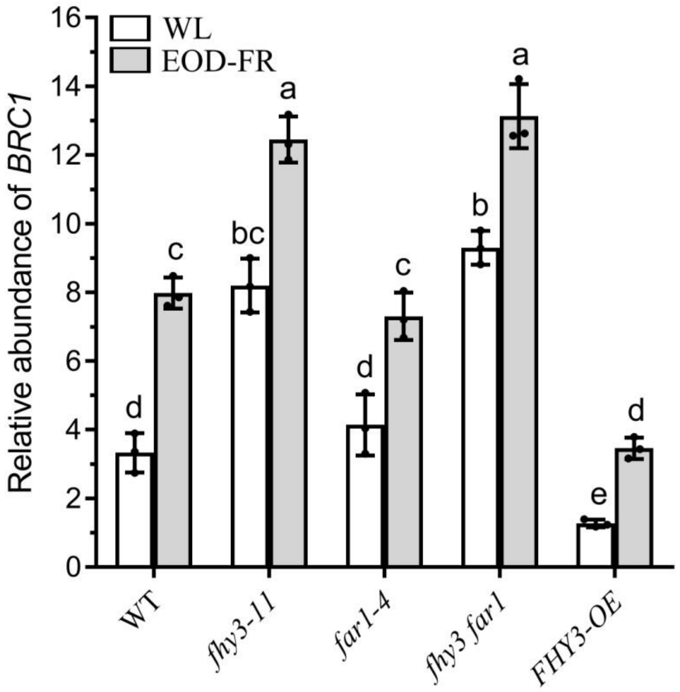

Supplementary Figure 1. Comparison of *BRC1* expression levels in the *fhy3* loss-of-function mutant and *FHY3*-OE plants with or without EOD-FR treatments. Plants were grown under WL with or without EOD-FR treatment for four weeks and the axillary buds were harvested for RNA extraction. Values given are mean ± SD (n=3). Different letters indicate significant differences by the two-sided LSD test (p<0.05).

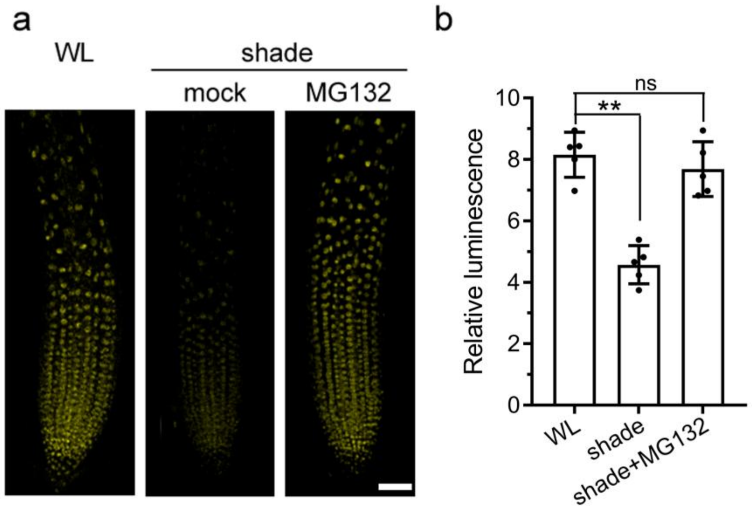

Supplementary Figure 2. Comparison of FHY3 protein levels in the *pFHY3::YFP-FHY3* transgenic seedlings grown under white light (WL) or treated with simulated shade (for 30 minutes) or MG132 (10 μM MG132 for 4 hours). The YFP fluorescence was visualized with Zeiss confocal microscope system. This assay was repeated for three times and similar results were obtained. Values shown are mean ± SD (n=5). ***p*<0.01 by the two-sided Student *t*-test; ns, no significant differences. Scale bar= 50 μm.

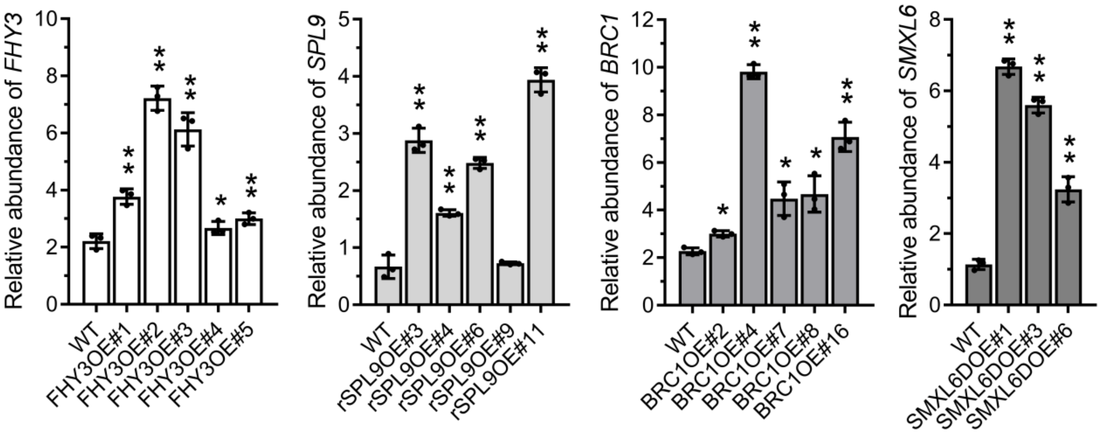

Supplementary Figure 3. RT-qPCR analysis of the relative expression levels of various transgenes in their respective overexpression lines. Axillary buds were used for total RNA extraction for measuring *BRC1* expression. For other genes, the leaves from three-week old plants were used. The transgenic lines *FHY3-OE#2*, *rSPL9-OE#11*, *BRC1-OE#4* and *SMXL6D-OE#1* were used in this study. **p*<0.05 and ***p*<0.01 by the two-sided Student *t*-test.

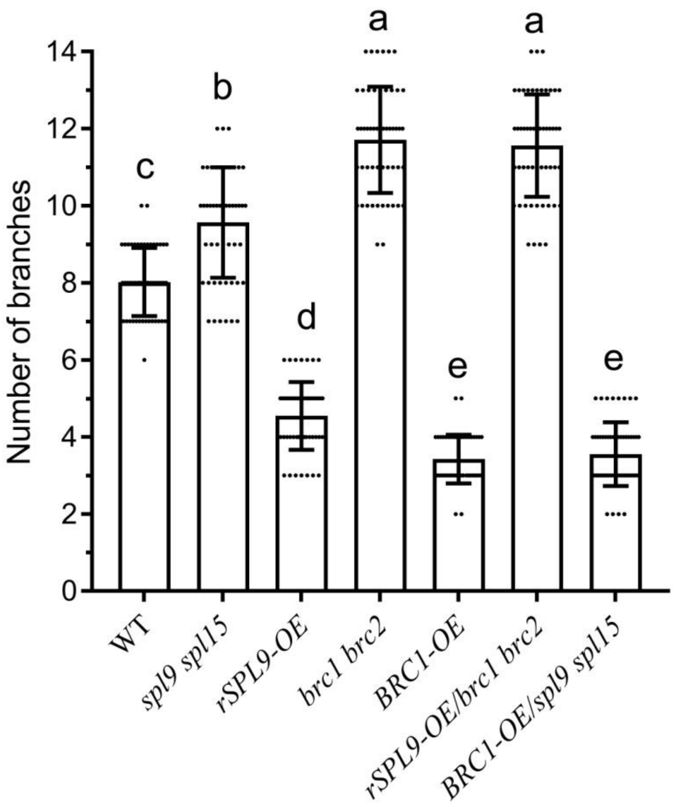

Supplementary Figure 4. Quantification of the rosette branch number of the plants shown in Fig. 2a. Values shown are mean ± SD (n>50). Different letters indicate significant differences by the two-sided LSD test (p<0.05).

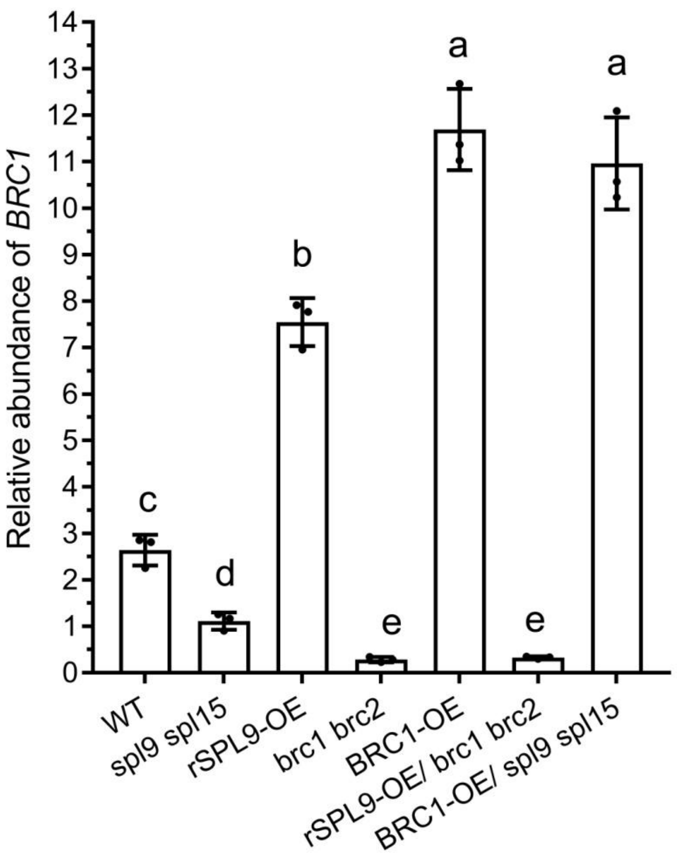

Supplementary Figure 5. Comparison of *BRC1* expression levels of the plants shown in Fig. 2a. Plants were grown under normal white light conditions for four weeks and the axillary buds were then harvested for RNA extraction. Values given are mean ± SD (n=3). Different letters indicate significant differences by the two-sided LSD test (p<0.05).

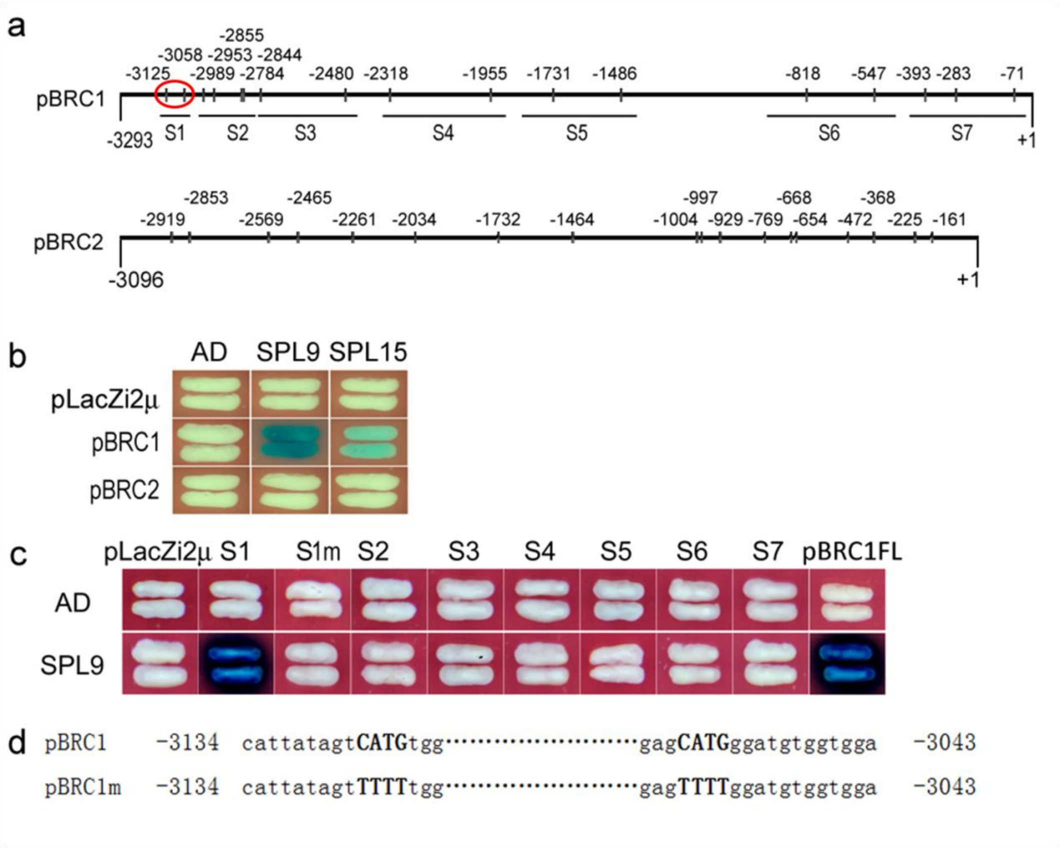

Supplementary Figure 6. Diagram of the *BRC1* and *BRC2* promoters and locations of the GTAC motifs. **a** The *BRC1* and *BRC2* promoters harbor more than a dozen core SPL binding sites (indicated by the black vertical lines and their positions are marked with corresponding numbers). The position of the start codon ATG is designed +1. Red cycle indicates the binding sites of the *BRC1* promoter for SPL9 and SPL15 confirmed in this study. **b** Yeast one hybrid assay shows that both SPL9 and SPL15 do not bind to the *BRC2* promoter. The binding of SPL9 or SPL15 to the *BRC1* promoter is used as a positive control. **c** Deletion analysis shows that SPL9 binds to the GTAC motifs in the S1 fragment of the *BRC1* promoter. The positions of S1 to S7 are shown in **a**. pBRC1FL represents the full-length *BRC1* promoter and S1m indicates the S1 fragment with mutated GTAC motifs. **d** A diagram showing mutagenesis of the GTAC motifs in the S1 fragment into TTTT.

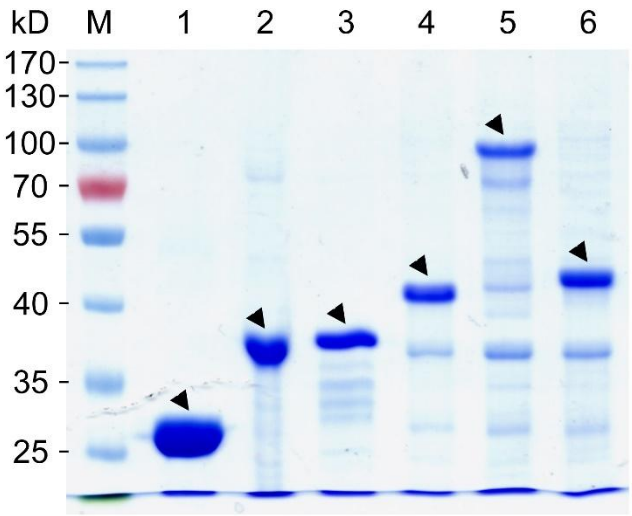

Supplementary Figure 7. SDS-PAGE analysis of purified recombinant GST, GST-SPL9SBP, GST-SPL15SBP, His-FHY3-I, His-SMXL6 and His-MBP proteins. M: molecular mass standard; Line 1: GST; Line 2: GST-SPL9SBP domain proteins; Line 3: GST-SPL15SBP domain proteins; Line 4: His-FHY3-I proteins; Line 5: His-SMXL6 proteins; Line 6: His-MBP proteins. The triangles indicate the target proteins.

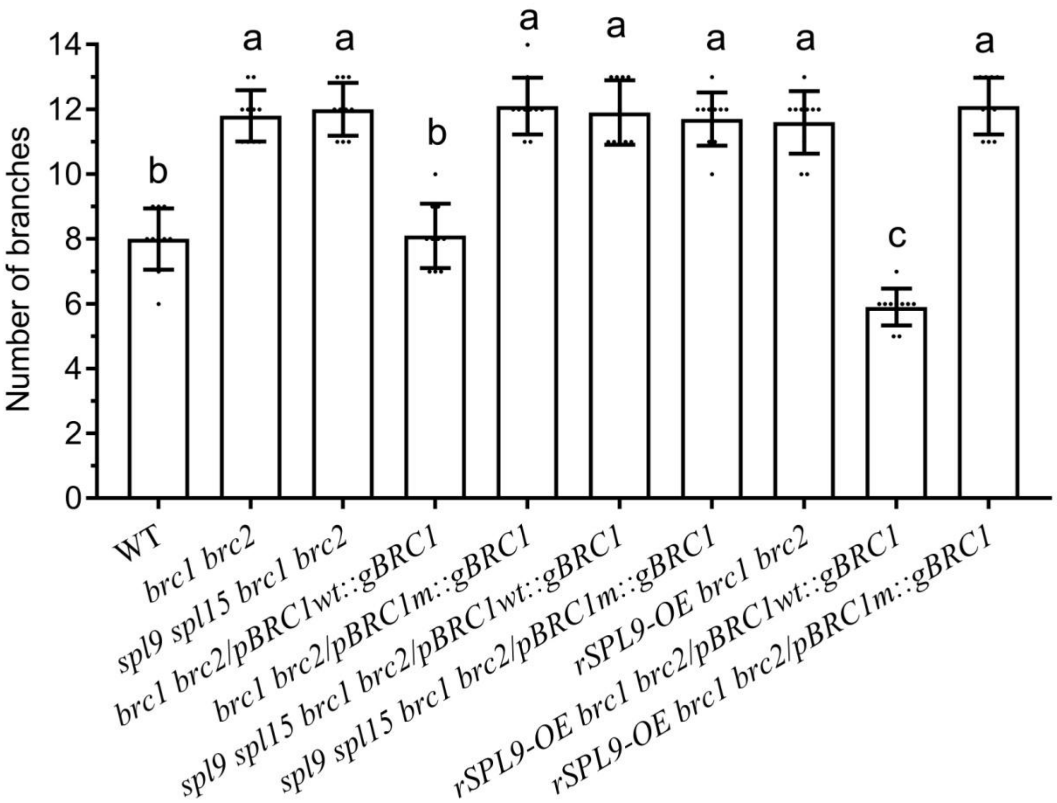

Supplementary Figure 8. Comparison of the rosette branch number of the transgenic plants expressing *BRC1* driven by its endogenous wild type promoter (*pBRC1wt::gBRC1*) or *BRC1* promoter mutated in the SBP binding site (*pBRC1m::gBRC1*). Ten independent T_1 transgenic lines harboring the *pBRC1wt::gBRC1* or *pBRC1m::gBRC1* transgene were used for statistic analysis. Values shown are mean ± SD (n=10). Different letters indicate significant differences by the two-sided LSD test (p<0.05).

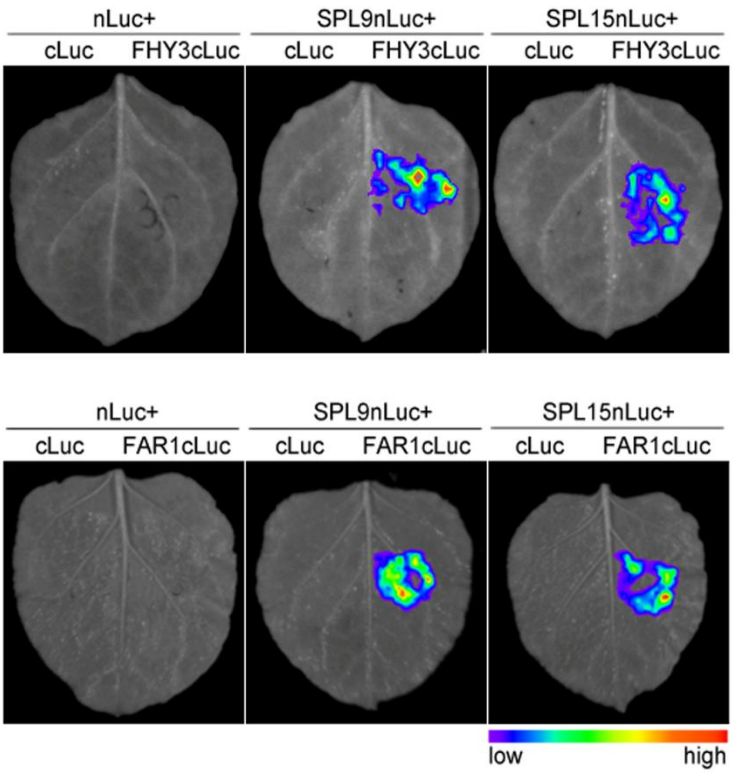

Supplementary Figure 9. FHY3 and FAR1 directly interact with SPL9 and SPL15 by LCI assay. SPL9 and SPL15 were fused to the N-terminal fragment of luciferase (nLuc) while both FHY3 and FAR1 were fused to the C-terminal fragment of luciferase (cLuc). The interactions between cLuc and SPL9-nLuc or SPL15-nLuc are used as negative controls. Representative images of *N. benthamiana* leaves 72 h after infiltration are shown.

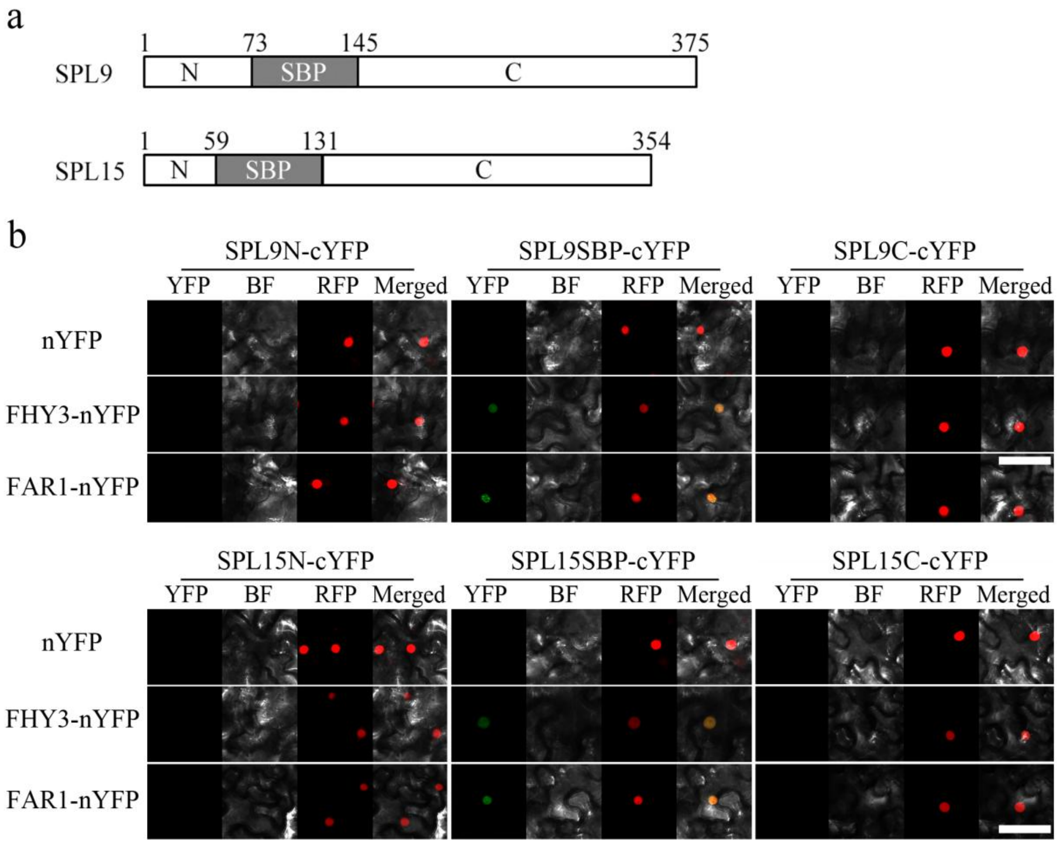

Supplementary Figure 10. BiFC assay shows that the SBP domains of SPL9 and SPL15 are required for interacting with FHY3 and FAR1. a Schematic diagram of SPL9 and SPL15 proteins. N, N-terminal; C, C-terminal. **b** BiFC assay shows that the SBP domains of SPL9 and SPL15 are required for their interaction with FHY3 and FAR1 proteins. Scale bar = 50 μm.

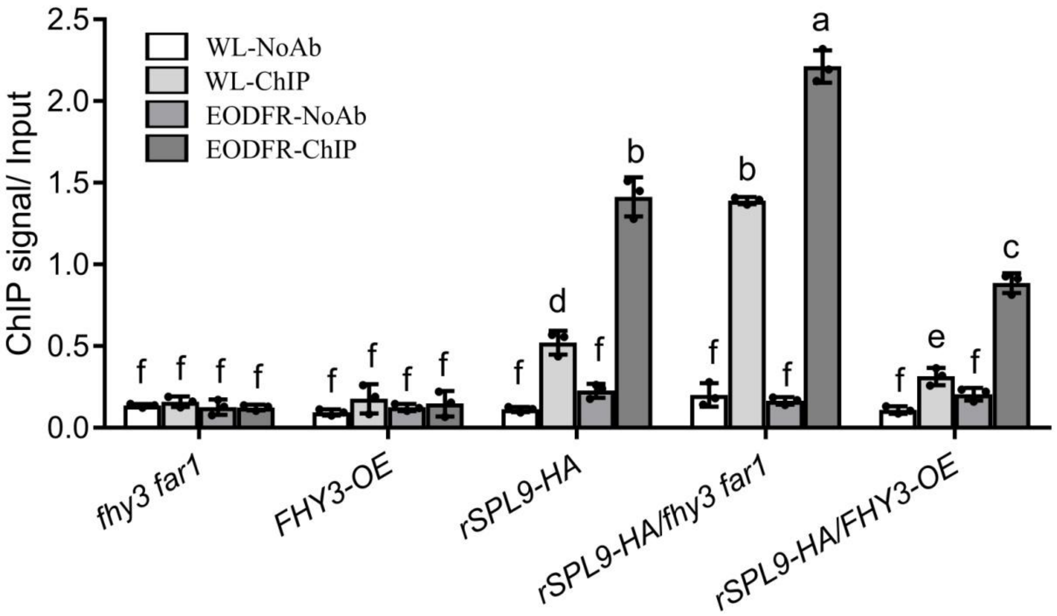

Supplementary Figure 11. ChIP-qPCR analysis using the *p35S::rSPL9-HA* transgenic seedlings shows that the enrichment of *BRC1* promoter fragments by SPL9 is inhibited by the presence of FHY3. Ten-day-old seedlings grown under normal white light conditions with or without EOD-FR treatments were harvested for RNA extraction. Values shown are mean ± SD (n=3). Letters indicate significant differences by the two-sided LSD test (p<0.05).

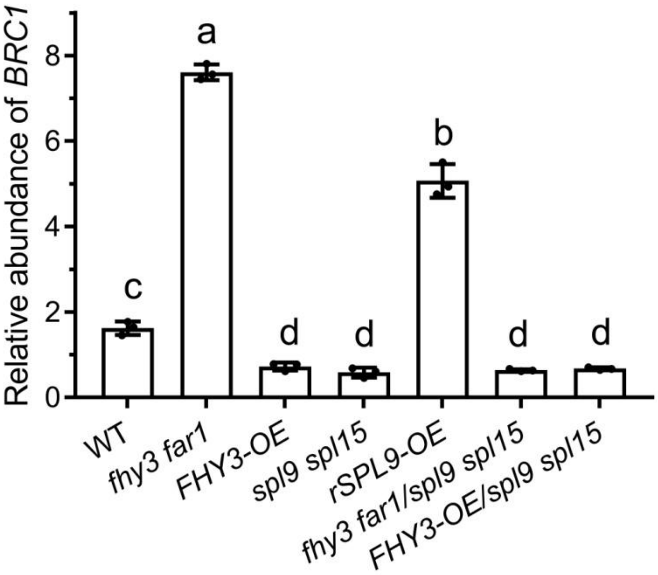

Supplementary Figure 12. Comparison of *BRC1* expression levels of the plants shown in Fig. 4e. Plants were grown under normal white light conditions for four weeks and the axillary buds were harvested for RNA extraction. Values given are mean ± SD (n=3). Different letters indicate significant differences by the two-sided LSD test (p<0.05).

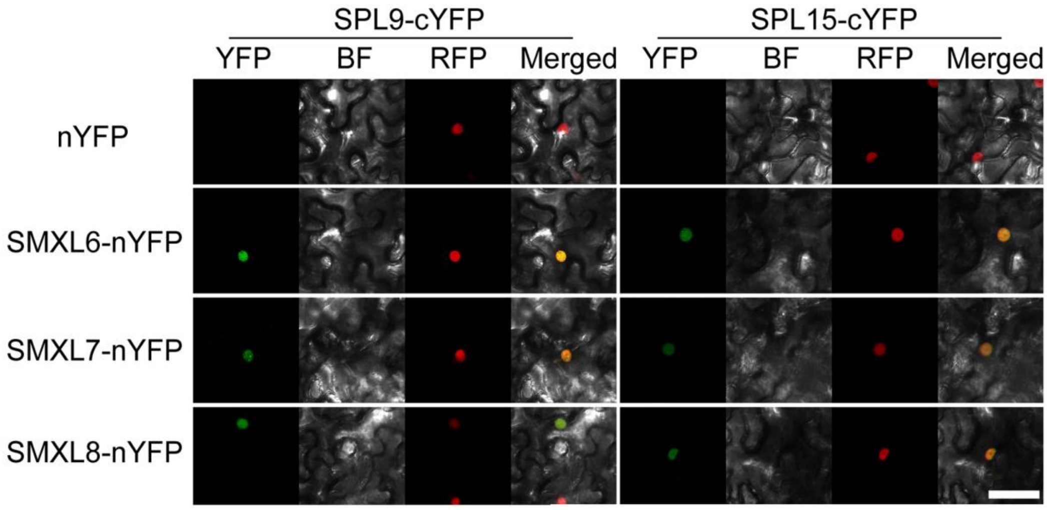

Supplementary Figure 13. BiFC assay shows that SMXL6/7/8 directly interact with SPL9 and SPL15. SPL9 and SPL15 were fused to the C-terminal fragment of YFP (cYFP) while SMXL6/7/8 were fused to the N-terminal fragment of YFP (nYFP). The interactions between nYFP and SPL9-cYFP or SPL15-YFP are used as negative controls. Representative images of *N. benthamiana* leaves 72 h after infiltration are shown. Scale bar= 50 μm.

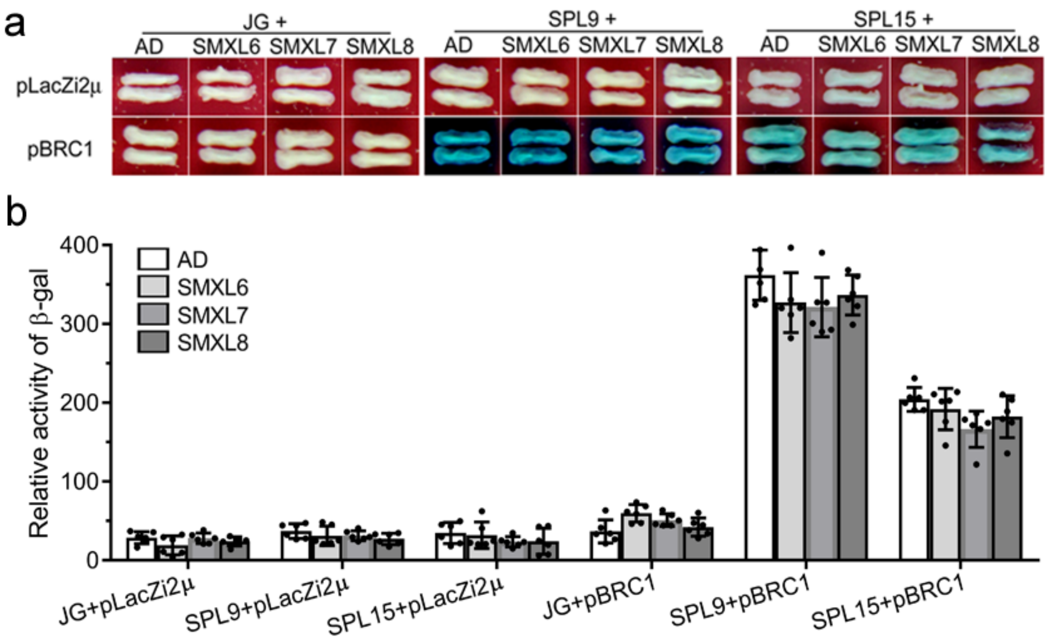

Supplementary Figure 14. Yeast one-hybrid assay shows that SMXL6/7/8 do not bind to the *BRC1* promoter and that they do not affect the DNA binding activity of SPL9/15. a Plate assay; **b** Liquid assay.

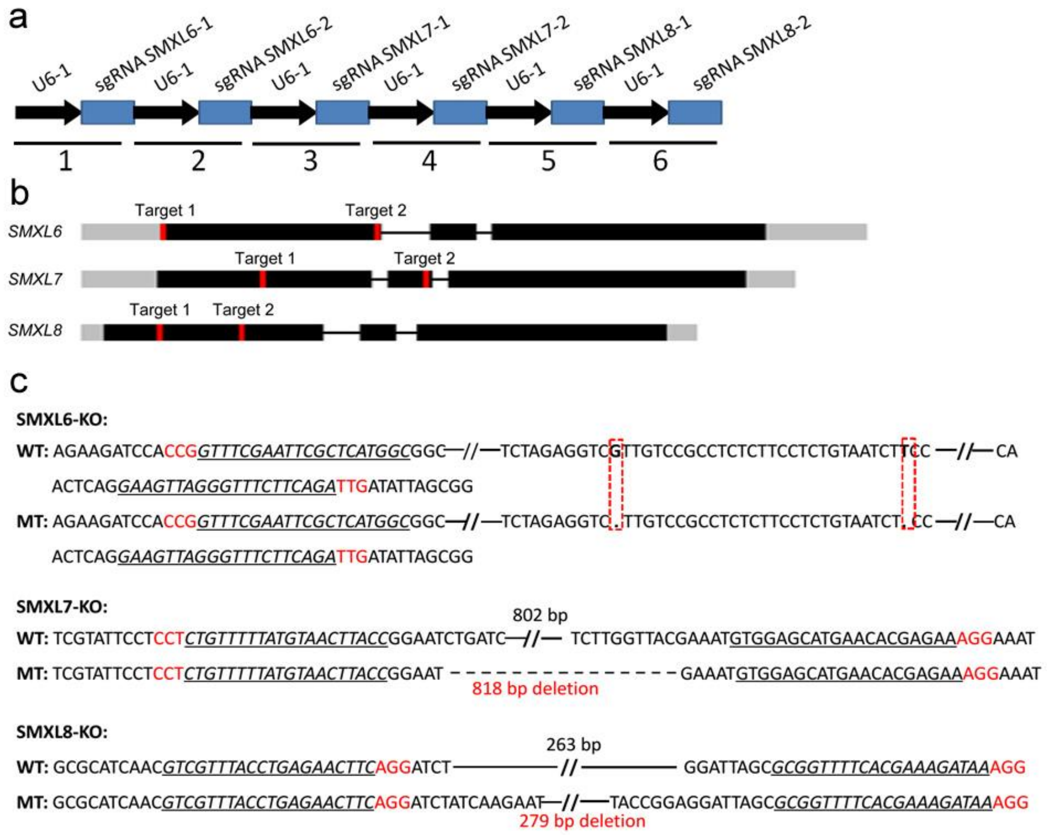

Supplementary Figure 15. Generation of *smxl6/7/8* triple mutant using CRISPR/Cas9 technology. a Diagram illustrating the sgRNA expression cassettes targeting *SMXL6*, *SMXL7* and *SMXL8* via the dual-sgRNAs CRISPR/Cas9 vector system. The primer pairs U6-1-1F /sgR-R and U6-1-2F /sgR-R were used to assemble the first sgRNA expression cassette (1) and the others (2, 3, 4, 5 and 6), respectively, step by step. **b** Diagram showing the two target sites on *SMXL6*, *SMXL7* and *SMXL8* genes. Gray boxes, black boxes and black lines indicate the untranslated regions, exons and introns, respectively. The target sites are indicated by red boxes. **c** Diagram showing the mutations of *SMXL6*, *SMXL7* and *SMXL8* genes in the *smxl6/7/8* triple knockout mutant used in this study. The target sequences are in italic and underlined. Dashed lines indicate multiple nucleotides deletion.

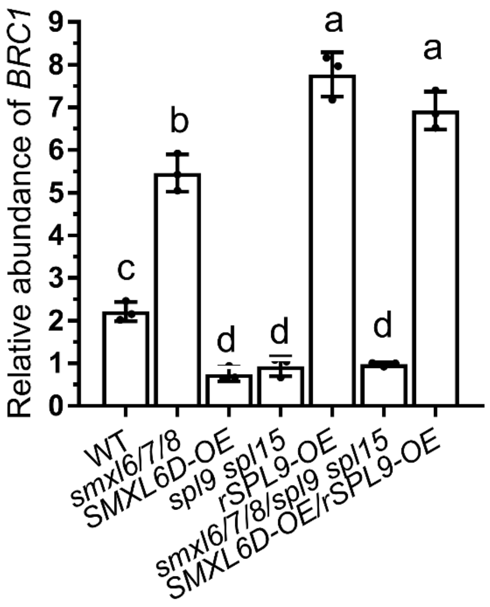

Supplementary Figure 16. Comparison of *BRC1* expression levels of the plants shown in Fig. 6a. Plants were grown under normal white light conditions for four weeks and the axillary buds were harvested for RNA extraction. Values given are mean ± SD (n=3). Different letters indicate significant differences by the two-sided LSD test (p<0.05).

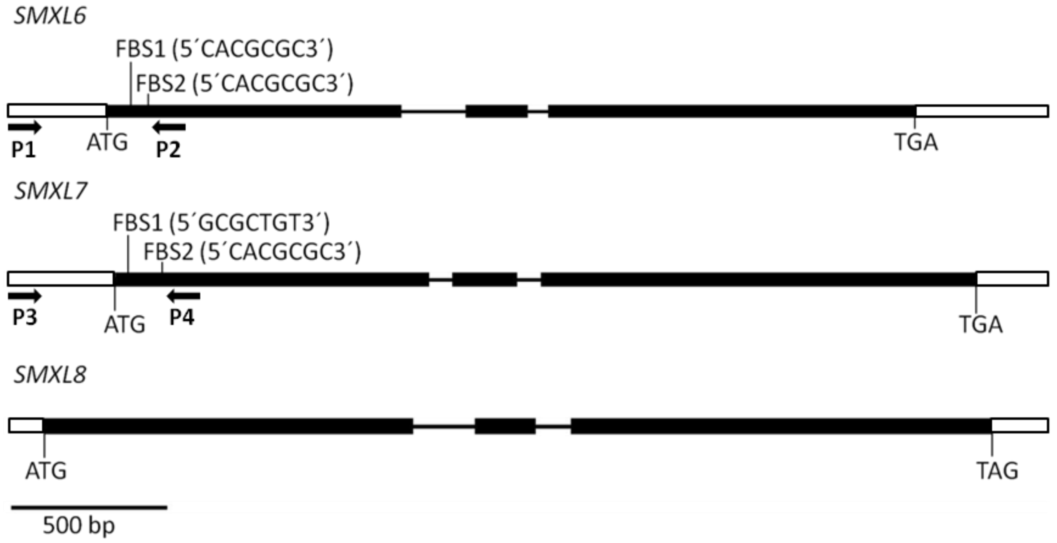

Supplementary Figure 17. Diagram of the gene structure of *SMXL6/7/8* and the positions of predicted FBS sites in the first exon of *SMXL6* and *SMXL7*. The DNA fragments used in the yeast one hybrid assay are indicated. Black boxes indicate exons. White boxes indicate the untranslated regions. The ATG start codon and TGA/TAG stop codons of these proteins are indicated. The fragments amplified with primer pair P1/P2 (for *SMXL6*) or P3/P4 (for *SMXL7*) were used for yeast one-hybrid assay in this study.

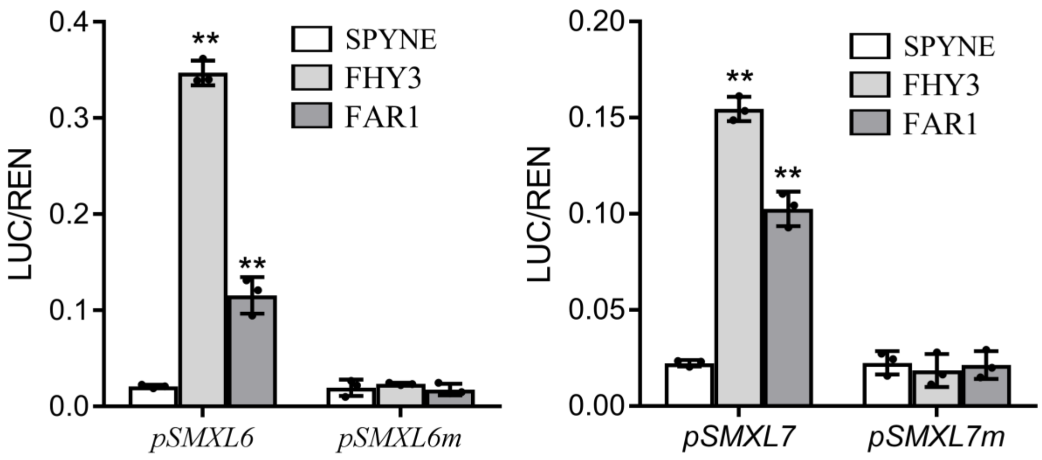

Supplementary Figure 18. Dual-luciferase assay of LUC expression in Fig. 6d. The expression of REN was used as an internal control. LUC/REN ration represents the relative activity of the promoters. Values given are mean ± SD (n = 3). **$p < 0.01$ by the two-sided Student’s *t*-test.

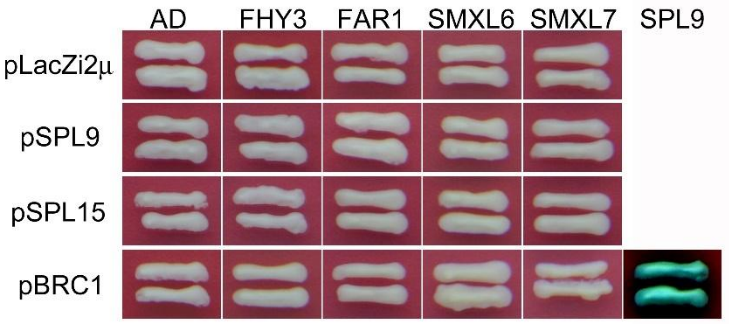

Supplementary Figure 19. Yeast one-hybrid assay shows that there is no direct binding of FHY3/FAR1 or SMXL6/7 to the promoters of *SPL9*, *SPL15* and *BRC1* genes. The binding of SPL9 to the *BRC1* promoter is used as a positive control.

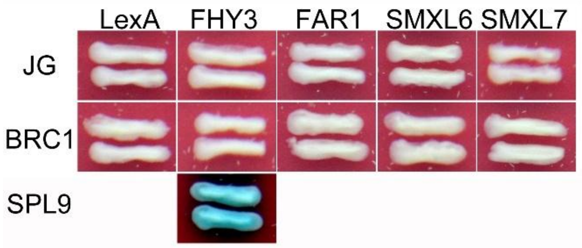

Supplementary Figure 20. Yeast two-hybrid assay shows that there is no direct physical interaction between FHY3/FAR1/SMXL6/SMXL7 and BRC1 proteins. The interaction between FHY3 and SPL9 is used as a positive control.

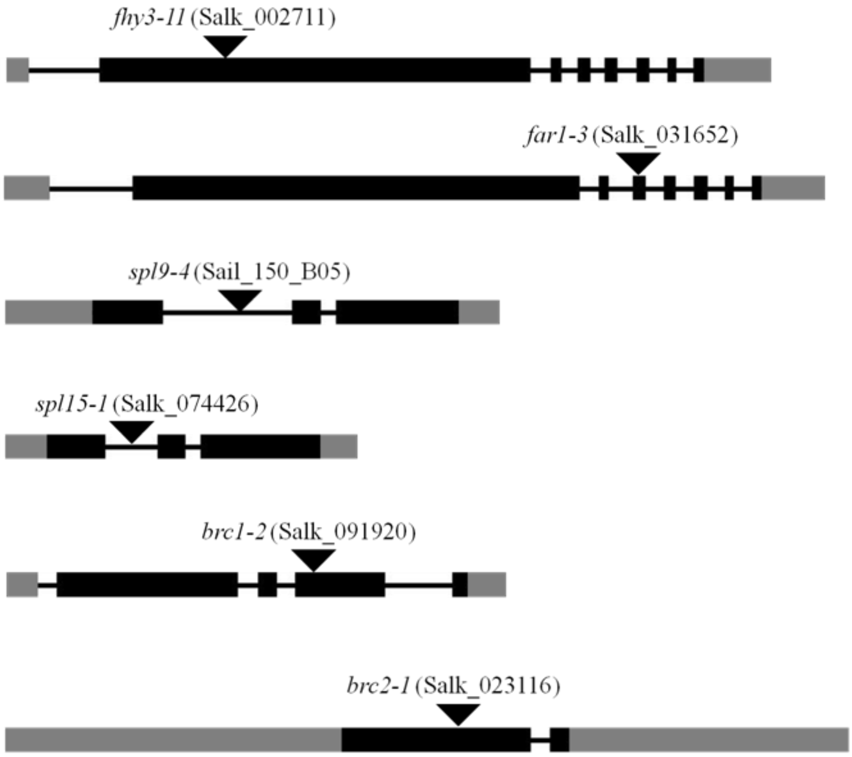

Supplementary Figure 21. Mutant alleles characterized in this study. Black boxes and black lines represent exons and intron, respectively. The gray boxes represent UTRs. The position of the T-DNA insertion is indicated by a triangle.

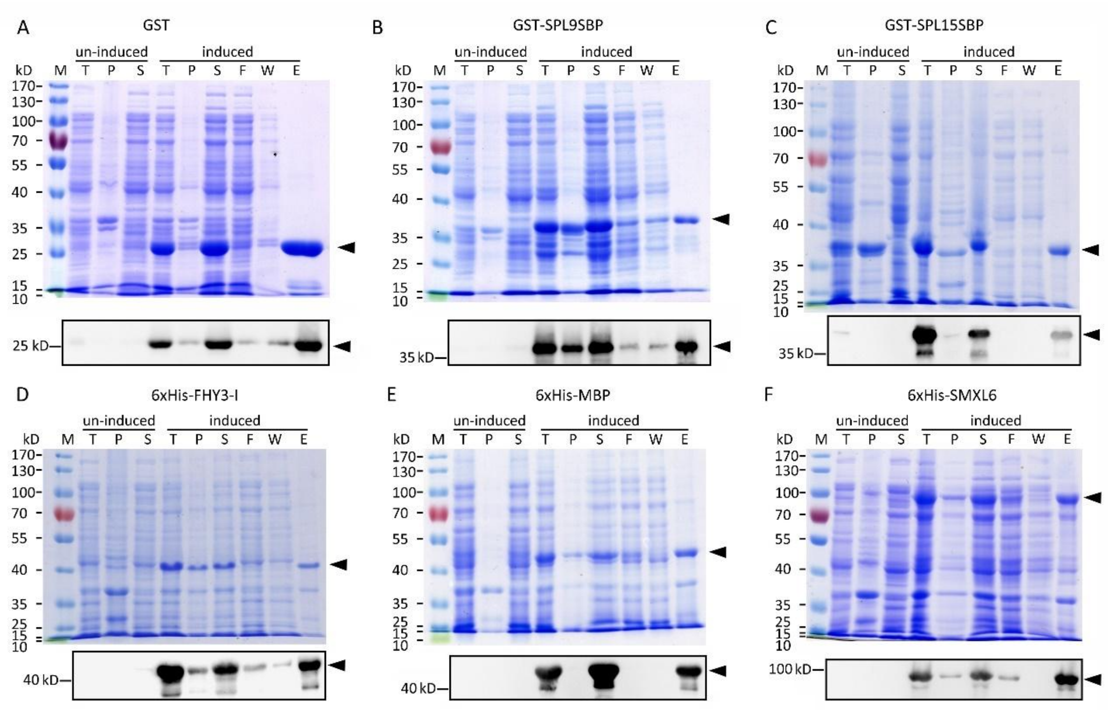

Supplementary Figure 22. Purification and western blotting of the recombinant proteins used in this study. M: molecular mass standard; T: total proteins; P: pellets; S: supernatants; F: flow through; W: wash fragments; E: elution fragments. The triangles indicate the target proteins.

A. SMXL6 (AT1G07200)

```
1     MPTPVTTARE CLTEEAARAL DDAVVVARRR SHAQTTSLHA VSALLAMPSS ILREVCVSRA
61    ARSVPYSSRL QFRALELCVG VSLDRLPSSK SPATEEDPPV SNSLMAAIKR SQANQRRHPE
121   SYHLQQIHAS NNGGGGCQTT VLKVELKYFI LSILDDPIVN RVFGEAGFRS SEIKLDVLHP
181   PVTQLSSRFS RGRCPPLFLC NLPNSDPNRE FPFSGSSGFD ENSRRIGEVL GRKDKKNPLL
241   IGNCANEALK TFTDSINSGK LGFLQMDISG LSLISIEKEI SEILADGSKN EEEIRMKVDD
301   LGRTVEQSGS KSGIVLNLGE LKVLTSEANA ALEILVSKLS DLLKHESKQL SFIGCVSSNE
361   TYTKLIDRFP TIEKDWDLHV LPITASTKPS TQGVYPKSSL MGSFVPFGGF FSSTSNFRVP
421   LSSTVNQTLS RCHLCNEKYL QEVAAVLKAG SSLSLADKCS EKLAPWLRAI ETKEDKGITG
481   SSKALDDANT SASQTAALQK KWDNICQSIH HTPAFPKLGF QSVSPQFPVQ TEKSVRTPTS
541   YLETPKLLNP PISKPKPMED LTASVTNRTV SLPLSCVTTD FGLGVIYASK NQESKTTREK
601   PMLVTLNSSL EHTYQKDFKS LREILSRKVA WQTEAVNAIS QIICGCKTDS TRRNQASGIW
661   LALLGPDKVG KKKVAMTLSE VFFGGKVNYI CVDFGAEHCS LDDKFRGKTV VDYVTGELSR
721   KPHSVVLLEN VEKAEFPDQM RLSEAVSTGK IRDLHGRVIS MKNVIVVVTS GIAKDNATDH
781   VIKPVKFPEE QVLSARSWKL QIKLGDATKF GVNKRKYELE TAQRAVKVQR SYLDLNLPVN
841   ETEFSPDHEA EDRDAWFDEF IEKVDGKVTF KPVDFDELAK NIQEKIGSHF ERCFGSETHL
901   ELDKEVILQI LAASWSSLSS GEEEGRTIVD QWMQTVLARS FAEAKQKYGS NPMLGVKLVA
961   SSSGLASGVE LPAKVDVIW
```

B. FHY3 (AT3G22170)

```
1     MDIDLRLHSG DLCKGDDEDR GLDNVLHNEE DMDIGKIEDV SVEVNTDDSV GMGVPTGELV
61    EYTEGMNLEP LNGMEFESHG EAYSFYQEYS RAMGFNTAIQ NSRRSKTTRE FIDAKFACSR
121   YGTKREYDKS FNRPRARQSK QDPENMAGRR TCAKTDCKAS MHVKRRPDGK WVIHSFVREH
181   NHELLPAQAV SEQTRKIYAA MAKQFAEYKT VISLKSDSKS SFEKGRTLSV ETGDFKILLD
241   FLSRMQSLNS NFFYAVDLGD DQRVKNVFWV DAKSRHNYGS FCDVVSLDTT YVRNKYKMPL
301   AIFVGVNQHY QYMVLGCALI SDESAATYSW LMETWLRAIG GQAPKVLITE LDVVMNSIVP
361   EIFPNTRHCL FLWHVLMKVS ENLGQVVKQH DNFMPKFEKC IYKSGKDEDF ARKWYKNLAR
421   FGLKDDQWMI SLYEDRKKWA PTYMTDVLLA GMSTSQRADS INAFFDKYMH KKTSVQEFVK
481   VYDTVLQDRC EEEAKADSEM WNKQPAMKSP SPFEKSVSEV YTPAVFKKFQ IEVLGAIACS
541   PREENRDATC STFRVQDFEN NQDFMVTWNQ TKAEVSCICR LFEYKGYLCR HTLNVLQCCH
601   LSSIPSQYIL KRWTKDAKSR HFSGEPQQLQ TRLLRYNDLC ERALKLNEEA SLSQESYNIA
661   FLAIEGAIGN CAGINTSGRS LPDVVTSPTQ GLISVEEDNH SRSAGKTSKK KNPTKKRKVN
721   PEQDVMPVAA PESLQQMDKL SPRTVGIESY YGTQQSVQGM VQLNLMGPTR DNFYGNQQTM
781   QGLRQLNSIA PSYDSYYGPQ QGIHGQGVDF FRPANFSYDI RDDPNVRTTQ LHEDASRHS
```

C. SPL9 (AT2G42200)

```
1     MEMGSNSGPG HGPGQAESGG SSTESSSFSG GLMFGQKIYF EDGGGGSGSS SSGGRSNRRV
61    RGGGSGQSGQ IPRCQVEGCG MDLTNAKGYY SRHRVCGVHS KTPKVTVAGI EQRFCQQCSR
121   FHQLPEFDLE KRSCRRRLAG HNERRRKPQP ASLSVLASRY GRIAPSLYEN GDAGMNGSFL
181   GNQEIGWPSS RTLDTRVMRR PVSSPSWQIN PMNVFSQGSV GGGGTSFSSP EIMDTKLESY
241   KGIGDSNCAL SLLSNPHQPH DNNNNNNNNN NNNNNTWRAS SGFGPMTVTM AQPPPAPSQH
301   QYLNPPWVFK DNDNDMSPVL NLGRYTEPDN CQISSGTAMG EFELSDHHHQ SRRQYMEDEN
361   TRAYDSSSHH TNWSL
```

D. SPL15 (AT3G57920)

```
1     MELLMCSGQA ESGGSSSTES SSLSGGLRFG QKIYFEDGSG SRSKNRVNTV RKSSTTARCQ
61    VEGCRMDLSN VKAYYSRHKV CCIHSKSSKV IVSGLHQRFC QQCSRFHQLS EFDLEKRSCR
121   RRLACHNERR RKPQPTTALF TSHYSRIAPS LYGNPNAAMI KSVLGDPTAW STARSVMQRP
181   GPWQINPVRE THPHMNVLSH GSSSFTTCPE MINNNSTDSS CALSLLSNSY PIHQQQLQTP
241   TNTWRPSSGF DSMISFSDKV TMAQPPPIST HQPPISTHQQ YLSQTWEVIA GEKSNSHYMS
301   PVSQISEPAD FQISNGTTMG GFELYLHQQV LKQYMEPENT RAYDSSPQHF NWSL
```

Supplementary Figure 23. Sequence matches of the recombinant proteins identified by LC/MS/MS and Q-TOF analyses and the protein sequence from the Arabidopsis protein database. The matched sequences are shown in red.

Supplementary Table 1. Primers Used in This Study

Primer ID	primer sequence (5'-3')	Purpose
U01	Cgagctcggtacccgggagcactcagggcatcaatgc	Cloning *BRC1* promoter into pLacZi2μ vector for Y1H
U02	Cagagcacatgcctcgaggccttttaggggttttg	
U03	Gaattcgagctcggtacccgggcattcaaagaatttgatggag	Cloning *BRC2* promoter into pLacZi2μ vector for Y1H
U04	Catacagagcacatgcctcgagtagattcaagagagaaatag	
U05	Gaattcgagctcggtacccgggctcttgtacatatccaagcg	Cloning *SMXLl6* promoter into pLacZi2μ vector for Y1H
U06	Ctcgaggtcgacagatccccggggagcgaattcgaaaccggtg	
U07	Gaattcgagctcggtacccgggtacatatctatcttcacaagc	Cloning *SMXL7* promoter into pLacZi2μ vector for Y1H
U08	Ctcgaggtcgacagatccccgggccatggatttgatgaagatg	
JG01	Attatgcctctcccgaattcatggagatgggttccaactcg	Cloning *SPL9* coding region into pJG4-5 vector for Y1H
JG02	Ttctcgagtcggccgaattctcagagagaccagttggtatg	
JG03	Attatgcctctcccgaattcatggagttgttaatgtgttcg	Cloning *SPL15* coding region into pJG4-5 vector for Y1H
JG04	Ttctcgagtcggccgaattctcaaagagaccaattgaaatg	
JG05	Attatgcctctcccgaattcatggatatagatcttcgactac	Cloning *FHY3* coding region into pJG4-5 vector for Y1H
JG06	Ttctcgagtcggccgaattcttacgagtgtctagacgcgtc	
JG07	Attatgcctctcccgaattcatggatttgcaagagaatctg	Cloning *FAR1* coding region into pJG4-5 vector for Y1H
JG08	Ttctcgagtcggccgaattcctatagctgccttgatgaac	
JG09	Attatgcctctcccgaattcatgccgacgccggtgactac	Cloning *SMXL6* coding region into pJG4-5 vector for Y1H
JG10	Ttctcgagtcggccgaattctcaccatatcacatccaccttc	
JG11	Attatgcctctcccgaattcatgccgacaccagtaaccac	Cloning *SMXL7* coding region into pJG4-5 vector for Y1H
JG12	Ttctcgagtcggccgaattctcagatcacttcgactctcgc	
JG13	Attatgcctctcccgaattcatgccaacggcggtgaatgtag	Cloning *SMXL8* coding region into pJG4-5 vector for Y1H
JG14	Ttctcgagtcggccgaattcctactgagattttacaaagaac	
EG01	Tcgcaacggcgactggctggaattcatgccgacgccggtgactac	Cloning *SMXL6* coding region into pEG202 vector for Y2H
EG02	Catggtcgacggatccccgggaattctcaccatatcacatccaccttc	
EG03	Tcgcaacggcgactggctggaattcatgccgacaccagtaaccac	Cloning *SMXL7* coding region into pEG202 vector for Y2H
EG04	Catggtcgacggatccccgggaattctcagatcacttcgactctcgc	
EG05	Tcgcaacggcgactggctggaattcatgccaacggcggtgaatgtag	Cloning *SMXL8* coding region into pEG202 vector for Y2H
EG06	Catggtcgacggatccccgggaattcctactgagattttacaaagaac	
Q01	Gcccttgcttatcatttcc	qPCR of *FAR1*
Q02	Acccttatcaacctgactgc	
Q03	Cgtcaccaactcaaggtctg	qPCR of *FHY3*
Q04	Aacatcctgctctgggttc	
Q05	Cacactgcgaagacaagatc	qPCR of *BRC1*
Q06	Gatggtggttaatcactggg	
Q07	Taacgtggccaaaatgatgc	qPCR of *PP2A*
Q08	Gttctccacaaccgcttggt	
Q09	Tcctctgtaatcttcccaactc	qPCR of *SMXL6*
Q10	Gggttcttcttatctttcctgc	
Q11	Tggttacgaaatgtggagc	qPCR of *SMXL7*
Q12	Gggaatccgagaggctaatac	

Q17	Ttggtgatatgatatatgttg	ChIP-qPCR of S1 fragment
Q18	Ctcttccctctctctctatctc	
Q19	Agataaagaagaaagagcatg	ChIP-qPCR of S2 fragment
Q20	Ataaaaactcctattttggtca	
Q21	Tgatagtgtttcatctttgc	ChIP-qPCR of S3 fragment
Q22	Taggtagttatgttgaccttg	
P01	ggtcgcggatccgaattcatggatatagatcttcgacta	Cloning *FHY3-I* into pET28a vector
P02	tcgacggagctcgaattcttaggaattcaaggattgcatccg	
P03	gttccgcgtggatccccggaattcatgataccaaggtgccaagtggaag	Cloning *SPL9* SBP domain into pGEX-4T-1 vector
P04	ccgctcgagtcgacccgggaattcttaaggctgtggcttccttcgtcg	
P05	gttccgcgtggatccccggaattcatgacggcgaggtgccaagtg	Cloning *SPL15* SBP domain into pGEX-4T-1 vector
P06	ccgctcgagtcgacccgggaattcttattgtggttttcttcgtcgttc	
P07	ggtcgcggatccgaattcatgaaaatcgaagaaggtaaac	Cloning *MBP* into pET28a vector
P08	tcgacggagctcgaattcttagtgatggtgatggtgatggtg	
P09	ggtcgcggatccgaattcatgccgacgccggtgactac	Cloning *SMXL6* into pET28a vector
P10	tcgacggagctcgaattctcaccatatcacatccaccttc	
PB01	gaagaagccagcgagtccaaccattatagtcatgtggtcctcctgataattgcaggccta	Probe for *BRC1* EMSA
PB02	taggcctgcaattatcaggaggaccacatgactataatggttggactcgctggcttcttc	
M01	gaagaagccagcgagtccaaccattatagttttttggtcctcctgataattgcaggccta	Mutant probe for *BRC1* EMSA
M02	taggcctgcaattatcaggaggaccattttactataatggttggactcgctggcttcttc	
M03	cggttacgtaatccaccacgaatttgtcatcaagggaac	Mutagenesis of the *SMXL6 (SMXL6D)*
M04	gttcccttgatgacaaattcgtggtggattacgtaaccg	
M05	cgacctgcaggcatgcaagcttatgaacaacaacattttcag	Cloning the wild type *BRC1* promoter
M06	gtaccgagctcgaattcgcctttttaggggtttttgaag	
M07	cagcgagtccaaccattatagtcgcgggtcctcctgataattgcaggcctagagatagagagaga	Mutagenesis of SBP binding sites in *BRC1* promoter
M08	taattatccaccacatccgcgcctctttcttctttatctctcttccctctctctctatctctaggcctgc	
M09	ctgcaggcatgcaagcttatgaacaacaacattttcag	Cloning the *BRC1* coding region
M10	cgacggccagtgccaagcttatccactcgaactatgaaaac	
NE01	cgccactagtggatccatggatttgcaagagaatctg	Cloning *FAR1* into pSPYNE vector
NE02	gagcggtaccctcgagtagctgccttgatgaac	
NE03	cgccactagtggatccatggatatagatcttcgactac	Cloning *FHY3* into pSPYNE vector
NE04	gagcggtaccctcgagttacgagtgtctagacgcgtc	
CE01	cgccactagtggatccatggagatgggttccaactcgg	Cloning *SPL9* into pSPYCE vector
CE02	gagcggtaccctcgaggagagaccagttggtatggtg	
CE03	cgccactagtggatccatggagttgttaatgtgttc	Cloning *SPL15* into pSPYCE vector
CE04	gagcggtaccctcgagaaagagaccaattgaaatgttg	
NL01	ctcggtacccgggatccaatggatatagatcttcgactac	Cloning *FHY3* into pCAMBIA 1300-nLUC vector
NL02	gtacgagatctggtcgacttacgagtgtctagacgcgtc	
NL03	ctcggtacccgggatccaatggatttgcaagagaatctg	Cloning *FAR1* into pCAMBIA 1300-nLUC vector
NL04	gtacgagatctggtcgacctatagctgccttgatgaac	
CL01	gcgtcccggggcggtaccatggagatgggttccaactc	Cloning *SPL9* into pCAMBIA

CL02	tgttggatcccgggtacctcagagagaccagttggtatg	1300-cLUC vector
CL03	gcgtcccggggcggtaccatggagttgttaatgtgttc	Cloning *SPL15* into pCAMBIA 1300-cLUC vector
CL04	tgttggatcccgggtacctcaaagagaccaattgaaatg	
NL05	cgggggacgagctcggtaccatgccgacaccagtaaccac	Cloning *SMXL7* into pCAMBIA 1300-nLUC vector
NL06	acgagatctggtcgactcagatcacttcgactctcgc	
NL07	cgggggacgagctcggtaccatgccaacggcggtgaatgtag	Cloning *SMXL8* into pCAMBIA 1300-nLUC vector
NL08	acgagatctggtcgacctactgagattttacaaagaac	
NL09	cgggggacgagctcggtaccatgccgacgccggtgactac	Cloning *SMXL6* into pCAMBIA 1300-nLUC vector
NL10	acgagatctggtcgacccatatcacatccaccttc	
E01	cgacctgcaggcatgcaagcttatgaacaacaacattttcag	Cloning *BRC1* into pCAMBIA 1301 for overexpression
E02	aacgacggccagtgccaagctttcaatacatgttttgatagttg	
HA01	cgatagccatgggcgagctcatggatatagatcttcgactac	Cloning *FHY3* with HA tag into pCAMBIA 1300 vector
HA02	aatcgataccgtcggagctcagcgtaatctggaacatcgtatgggtacgagtgtctagacgcgtc	
HA03	ctgcggcagcggccgaattcatgccgacgccggtgactac	Cloning *SMXL6* with HA tag into pCAMBIA 1300 vector
HA04	aatcgataccgtcggagctcagcgtaatctggaacatcgtatgggtattctcgagcccggggaattcccatatcacatccaccttcg	
MYC01	cgatagccatgggcgagctcatggagatgggttccaactc	Cloning *SPL9* into pCAMBIA 1307 vector which harboring 6xMYC
MYC02	atagctttaaatcgataccgtcggagagaccagttggtatg	
MYC03	cgatagccatgggcgagctcatggagttgttaatgtgttc	Cloning *SPL15* into pCAMBIA 1307 vector which harboring 6xMYC
MYC04	atagctttaaatcgataccgtcgaagagaccaattgaaatg	
CR01	gccatgagcgaattcgaaaccggatatacagctagagtcgaagtagtgattgccatgagcgaattcgaaacgttttagagctagaaatagcaagtt	*SMXL6* knockout by CRISPR /Cas9 system
CR02	gaagttagggtttcttcagatggatatacagctagagtcgaagtagtgattgaagttagggtttcttcagagttttagagctagaaatagcaagtt	
CR03	ggtaagttacataaaaacagaggatatacagctagagtcgaagtagtgattggtaagttacataaaaacaggttttagagctagaaatagcaagtt	*SMXL7* knockout by CRISPR /Cas9 system
CR04	gtggagcatgaacacgagaaaggatatacagctagagtcgaagtagtgattgtggagcatgaacacgagaagttttagagctagaaatagcaagtt	
CR05	gtcgtttacctgagaacttcaggatatacagctagagtcgaagtagtgattgtcgtttacctgagaacttcgttttagagctagaaatagcaagtt	*SMXL8* knockout by CRISPR /Cas9 system
CR06	gcggttttcacgaaagataaaggatatacagctagagtcgaagtagtgattgcggttttcacgaaagataagttttagagctagaaatagcaagtt	
GT01	acggcgagagaatgcttgacg	Genotyping of s*mxl6*
GT02	gggataaaccccttgagtcgaag	
GT03	agatgacccgattgtgagtcg	Genotyping of *smxl7*
GT04	ggattctgcggtagagccacc	
GT05	cggtgaatgtagcgaaacaatg	Genotyping of *smxl8*
GT06	ccacactcttctcccgtgtcg	
