# Supplementary material for: Arabidopsis FHY3 and FAR1 integrate light and strigolactone signaling to regulate branching
Source: Nat Commun. 2020 Apr 23;11:1955. doi: 10.1038/s41467-020-15893-7 (PMC7181604; doi:10.1038/s41467-020-15893-7)

Supplementary images

1. **Root images of fluorescence microscopic analysis of the *pFHY3::YFP-FHY3* transgenic seedlings grown under normal white light (up panel) or simulated shade (down panel) conditions. The first images of each panel are used in Figure 1e. Scale bar= 50 μm.**

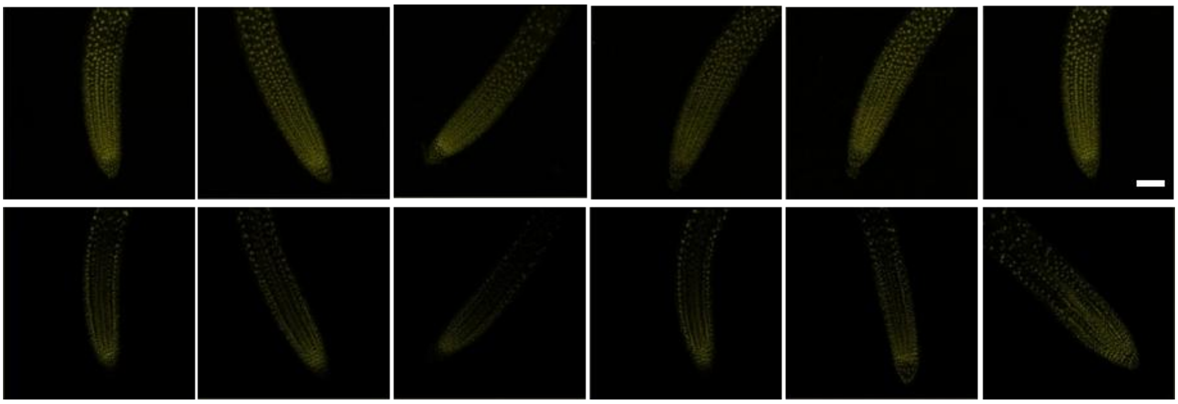

2. **Root images of fluorescence microscopic analysis of the *pFHY3::YFP-FHY3* transgenic seedlings grown under normal white light without (up panel), or with simulated shade treatment (middle panel), or with simulated shade treatment plus 10 μM MG132 (down panel). The first images of each panel are used in Supplementary Figure 2a. Scale bar=50 μm.**

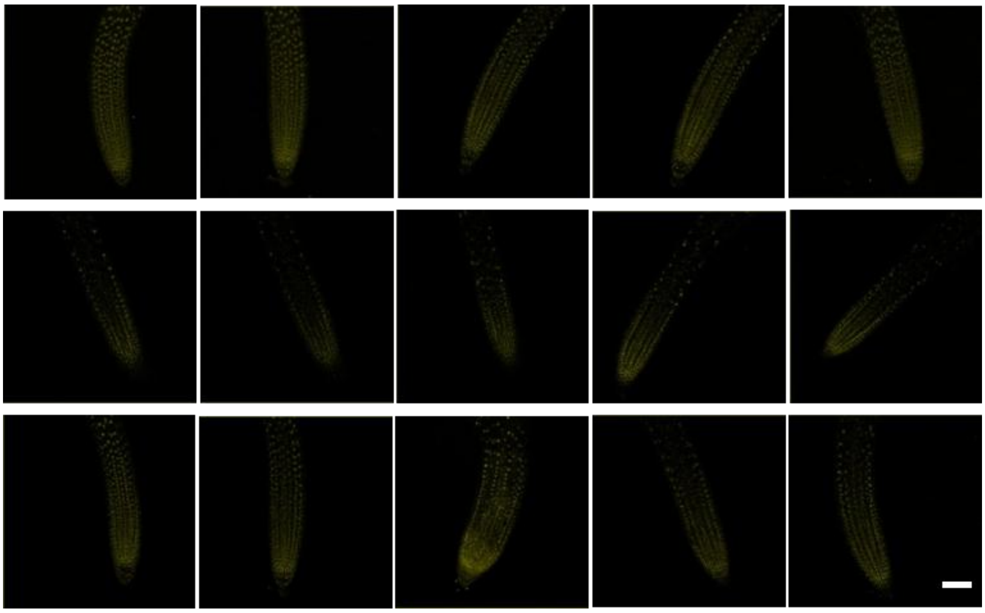

3. **Images for the luciferase complementation assays for the interactions between SPL9/15 with SMXL6/7/8 (four replicates each). There are two injected areas (left side and right side)**

in the leaf of each image. The control pairs of constructs were injected in the left side. The first images of each four replicates are used in Figure 5b.

Left: cLuc+nLuc; Right: cLuc+SPL9-nLuc

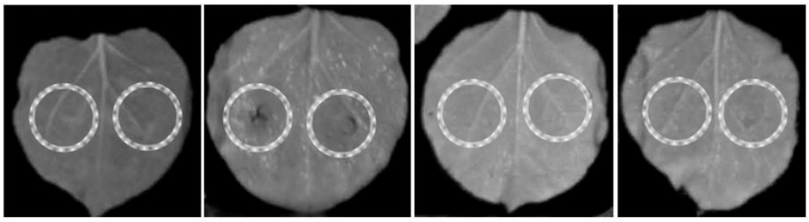

Left: SMXL6-cLuc+nLuc; Right: SMXL6-cLuc+SPL9-nLuc

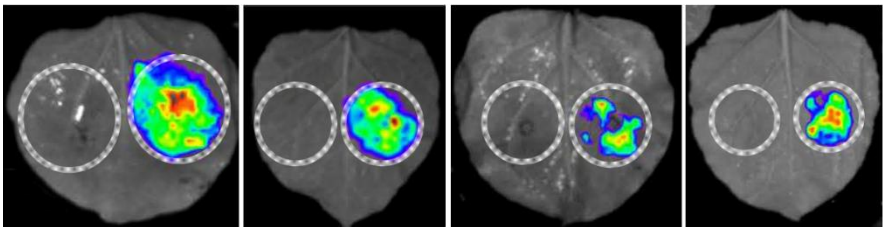

Left: SMXL7-cLuc+nLuc; Right: SMXL7-cLuc+SPL9-nLuc

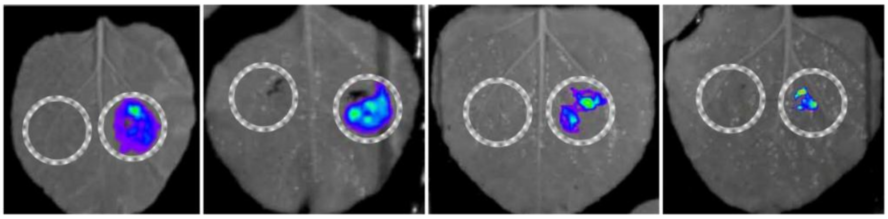

Left: SMXL8-cLuc+nLuc; Right: SMXL8-cLuc+SPL9-nLuc

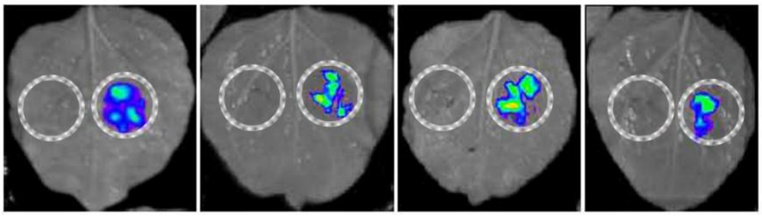

Left: cLuc+nLuc; Right: cLuc+SPL15-nLuc

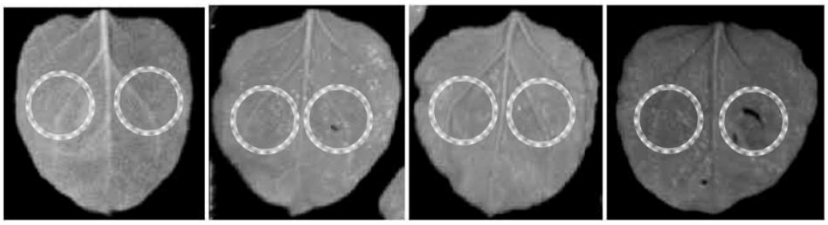

Left: SMXL6-cLuc+nLuc; Right: SMXL6-cLuc+SPL15-nLuc

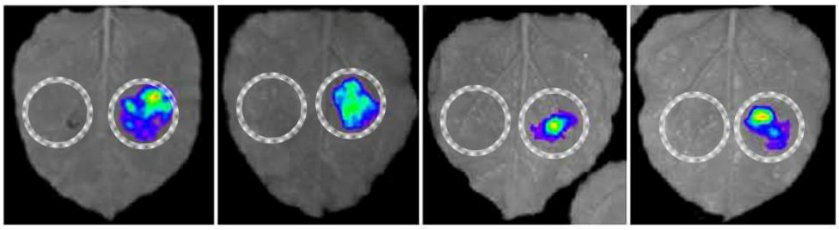

Left: SMXL7-cLuc+nLuc; Right: SMXL7-cLuc+SPL15-nLuc

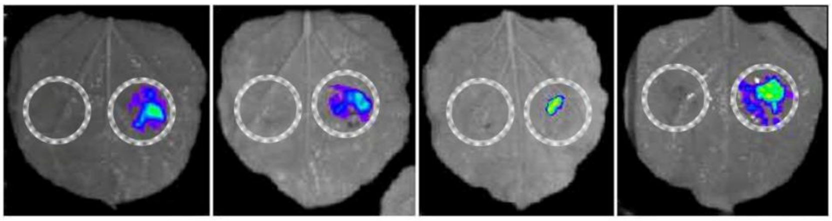

Left: SMXL8-cLuc+nLuc; Right: SMXL8-cLuc+SPL15-nLuc

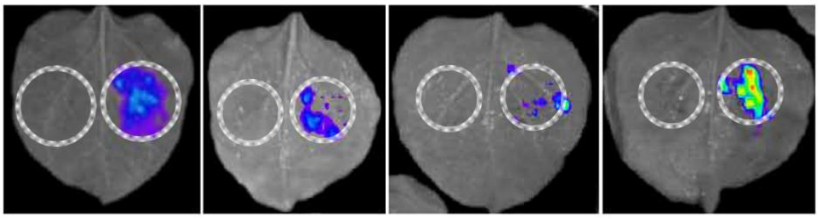

4. **Images for the luciferase complementation assays for the interactions between FHY3/FAR1 with SPL9/15 (four replicates each). There are two injected areas (left side and right side) in the leaf of each image. The control pairs of constructs were injected in the left side. The first images of each four replicates are used in Supplementary Figure 9.**

   Left: nLuc+cLuc; Right: nLuc+FHY3-cLuc

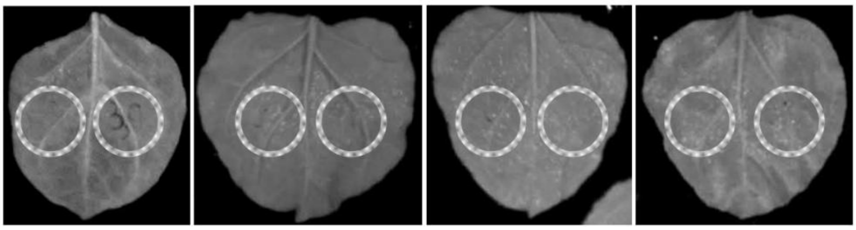

Left: SPL9-nLuc+cLuc; Right: SPL9-nLuc+FHY3-cLuc

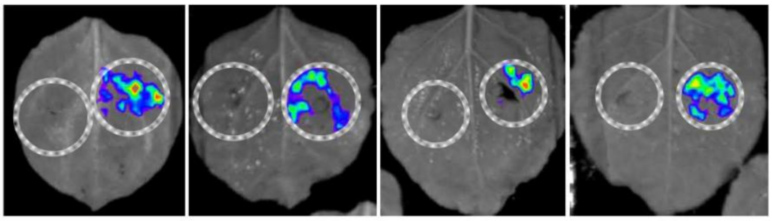

Left: SPL15-nLuc+cLuc; Right: SPL15-nLuc+FHY3-cLuc

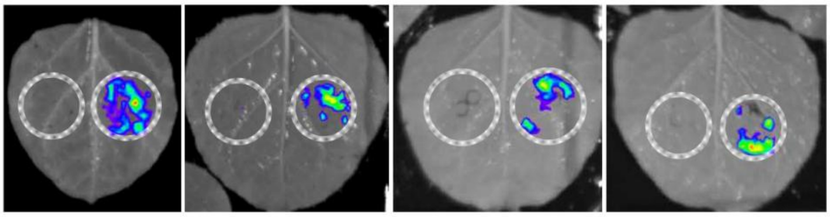

Left: nLuc+cLuc; Right: nLuc+FAR1-cLuc

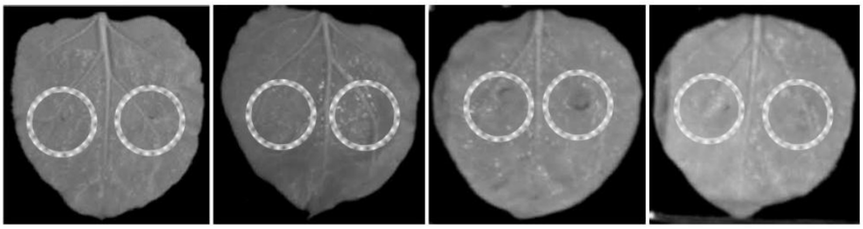

Left: SPL9-nLuc+cLuc; Right: SPL9-nLuc+ FAR1-cLuc

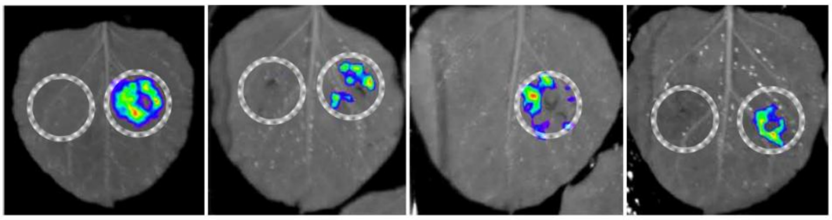

Left: SPL15-nLuc+cLuc; Right: SPL15-nLuc+ FAR1-cLuc

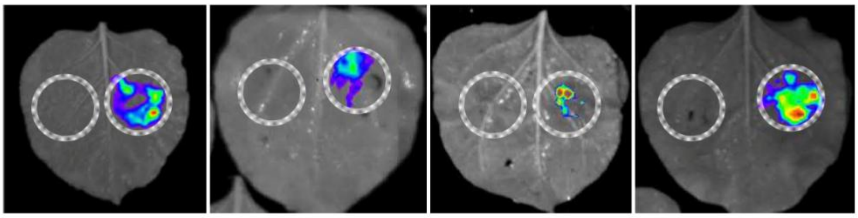

5. **The images of the transient expression assay for testing the effect of SPL9/15 on *pBRC1::LUC* and *pBRC1m::LUC* reporter genes (five replicates each). There are two injected areas (left side and right side) in the leaf of each image. The control pairs of constructs were injected in the left side. The first images of each panel are used in Figure 2e.**

Left: *SPYCE+pBRC1::LUC*; Right: SPL9+*pBRC1::LUC*

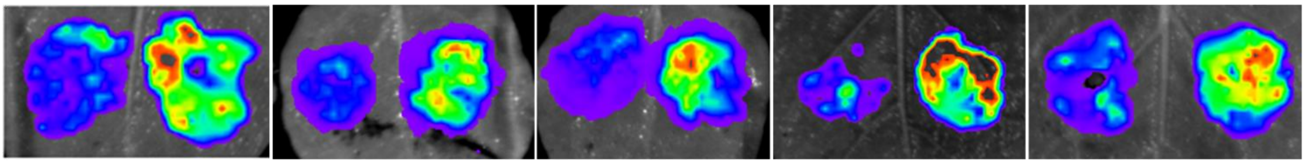

Left: *SPYCE+pBRC1m::LUC*; Right: SPL9+*pBRC1m::LUC*

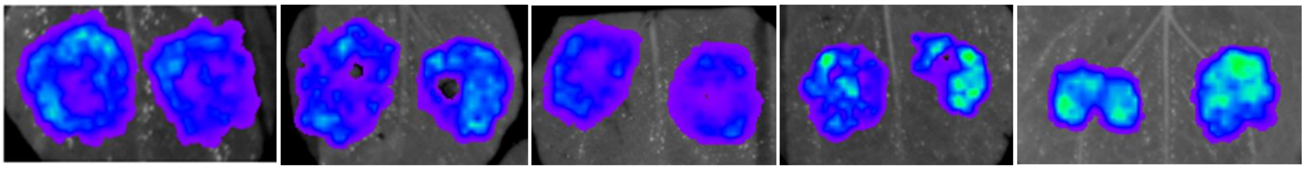

Left: *SPYCE+pBRC1::LUC*; Right: SPL15+*pBRC1::LUC*

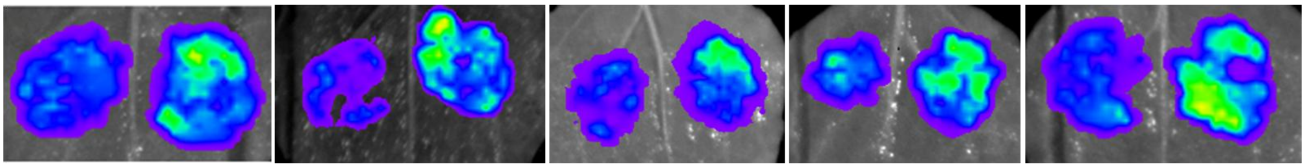

Left: *SPYCE+pBRC1m::LUC*; Right: SPL15+*pBRC1m::LUC*

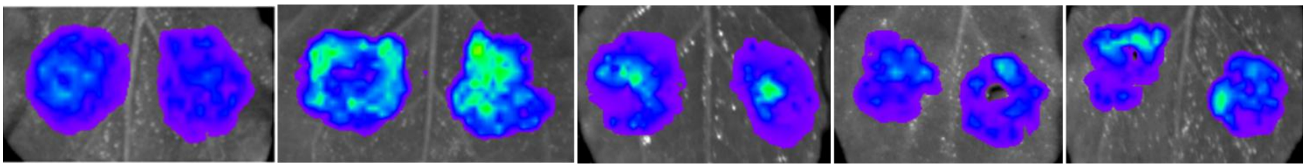

6. **The images of the transient expression assay for testing the effect of FHY3 on SPL9/15 activation of the *pBRC1::LUC* reporter gene (five replicates each). There are two injected areas (left side and right side) in the leaf of each image. The control pairs of constructs were injected in the left side. The first images of each panel are used in Figure 4d.**

Left: SPL9+ *SPYNE +pBRC1::LUC*; Right: SPL9+FHY3+*pBRC1::LUC*

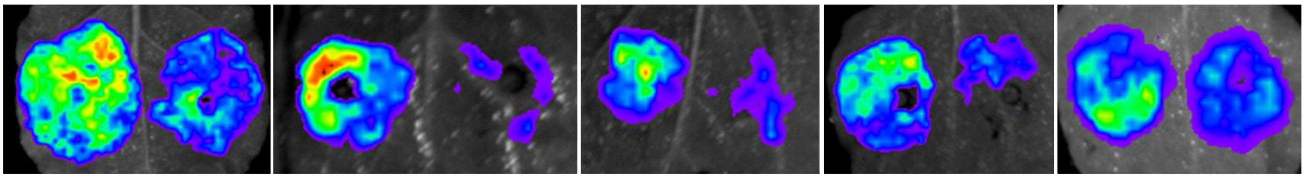

Left: SPL15+ *SPYNE +pBRC1::LUC*; Right: SPL15+FHY3+*pBRC1::LUC*

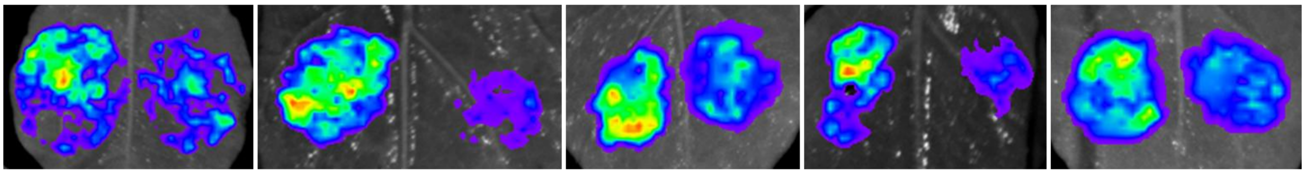

7. **The images of the transient expression assay for testing the effect of SMXL6/7/8 on SPL9/15 activation of the *pBRC1::LUC* reporter gene (five replicates each). There are two injected areas (left side and right side) in the leaf of each image. The control pairs of constructs were injected in the left side. The first images of each panel are used in Figure 5f .**

Left: SPL9+ *SPYNE +pBRC1::LUC*; Right: SPL9+SMXL6+*pBRC1::LUC*

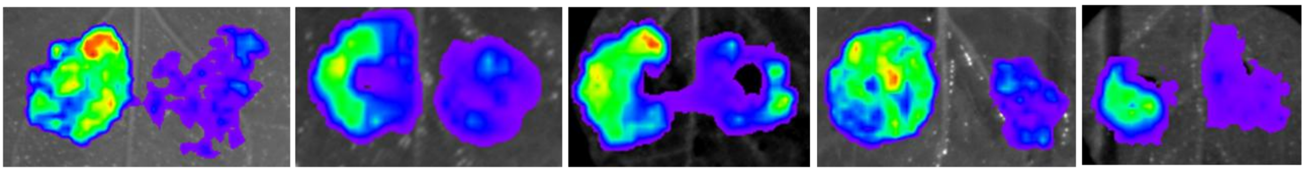

Left: SPL9+ *SPYNE* +*pBRC1::LUC*; Right: SPL9+SMXL7+*pBRC1::LUC*

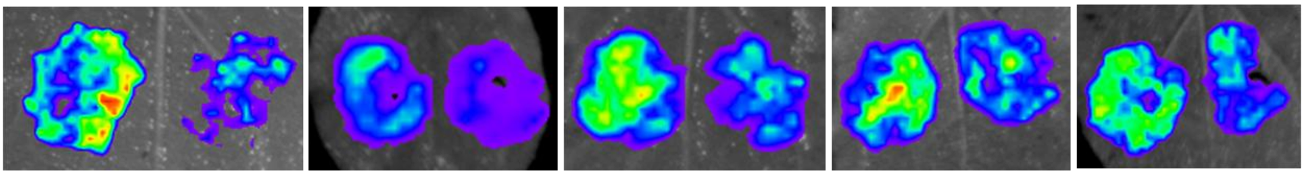

Left: SPL9+ *SPYNE* +*pBRC1::LUC*; Right: SPL9+SMXL8+*pBRC1::LUC*

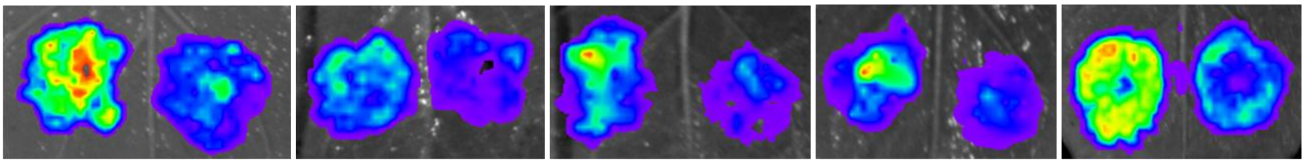

Left: SPL15+ *SPYNE* +*pBRC1::LUC*; Right: SPL15+SMXL6+*pBRC1::LUC*

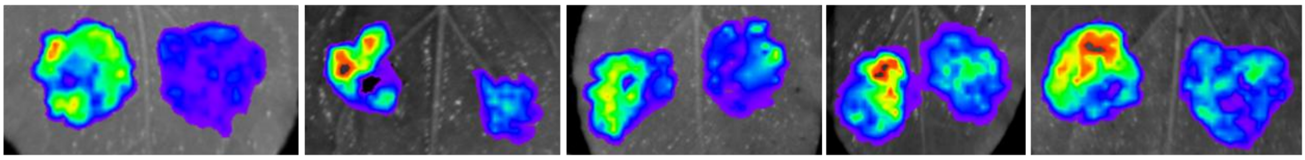

Left: SPL15+ *SPYNE* +*pBRC1::LUC*; Right: SPL15+SMXL7+*pBRC1::LUC*

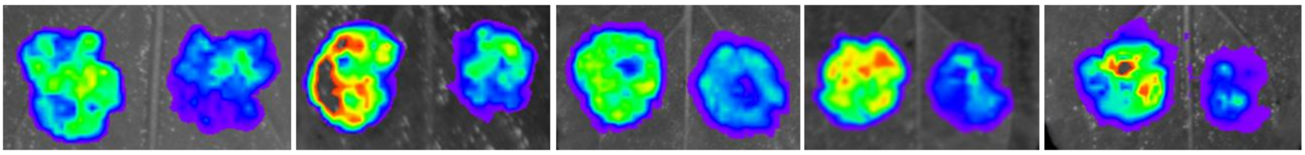

Left: SPL15+ *SPYNE* +*pBRC1::LUC*; Right: SPL15+SMXL8+*pBRC1::LUC*

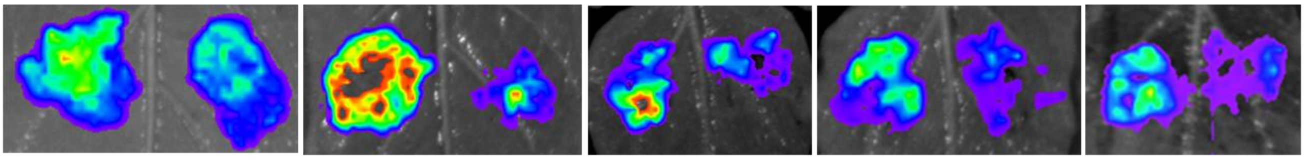

8. **The images for the transient expression assay for testing the effect of FHY3/FAR1 on SPL9/15 activation of the *pSMXL6/7::LUC* and *pSMXL6/7m::LUC* reporter genes (five replicates each). There are two injected areas (left side and right side) in the leaf of each image. The control pairs of constructs were injected in the left side. The first images of each panel are used in Figure 6d.**

   Left: *SPYNE* +*pSMXL6::LUC*; Right: FHY3+*pSMXL6::LUC*

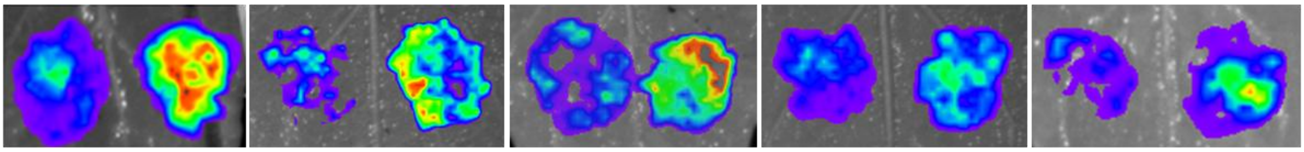

Left: *SPYNE +pSMXL6m::LUC*; Right: FHY3+*pSMXL6m::LUC*

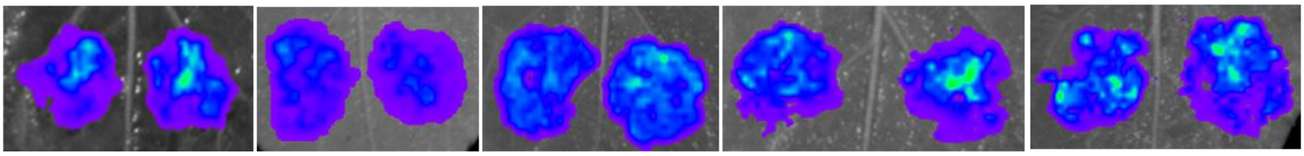

Left: *SPYNE +pSMXL6::LUC*; Right: FAR1+*pSMXL6::LUC*

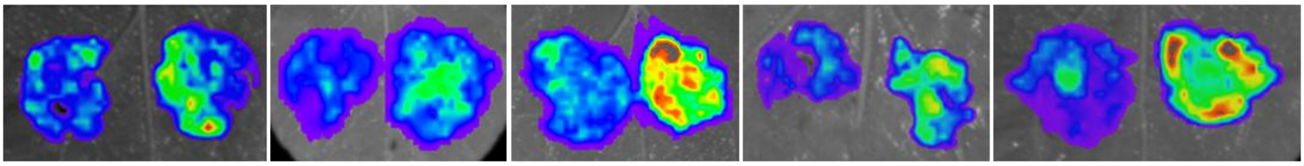

Left: *SPYNE +pSMXL6::LUC*; Right: FAR1+*pSMXL6::LUC*

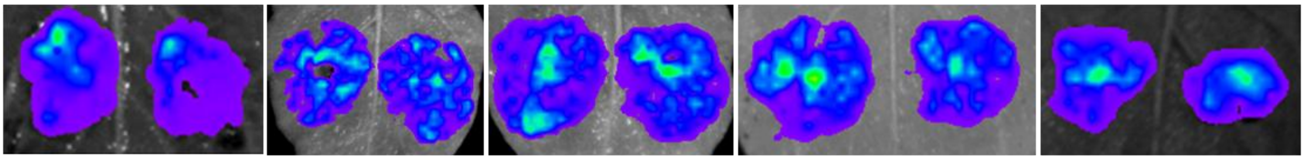

Left: *SPYNE +pSMXL7::LUC*; Right: FHY3+*pSMXL7::LUC*

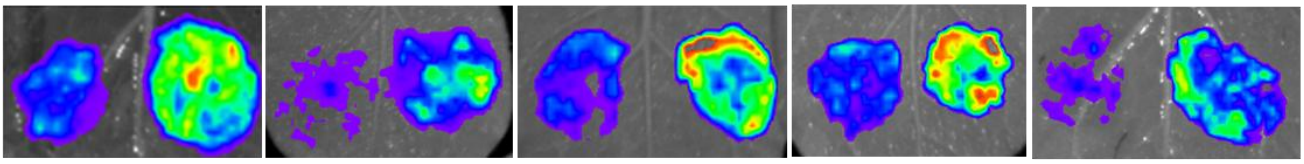

Left: *SPYNE +pSMXL7::LUC*; Right: FHY3+*pSMXL7m::LUC*

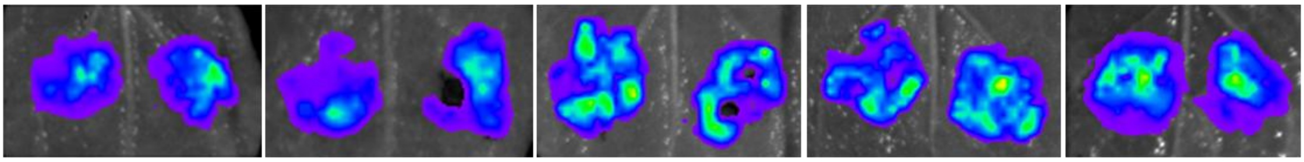

Left: *SPYNE +pSMXL7::LUC*; Right: FAR1+*pSMXL7::LUC*

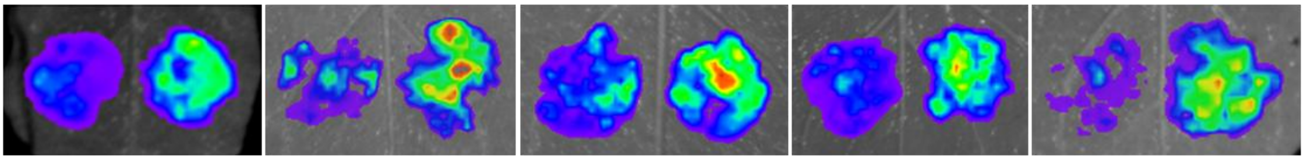

Left: *SPYNE +pSMXL7::LUC*; Right: FAR1+*pSMXL7m::LUC*

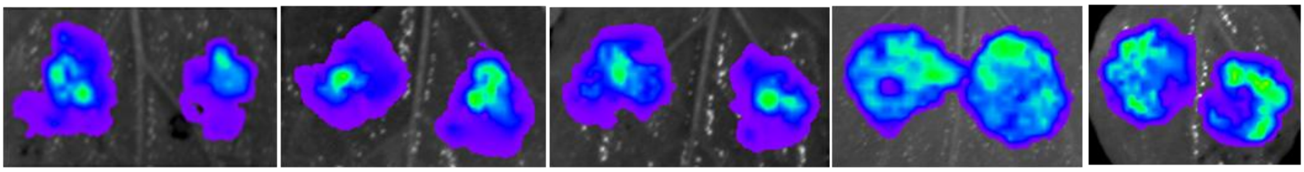

Supplement: Supplementary file 3 — Source Data [file 41467_2020_15893_MOESM3_ESM.zip › 213939_2_supp_4522273_q83gkp.pdf]
